# Psychological impact of COVID‐19 on hospital workers in nursing care hospitals

**DOI:** 10.1002/nop2.628

**Published:** 2020-09-20

**Authors:** Seoyon Yang, Sang Gyu Kwak, Min Cheol Chang

**Affiliations:** ^1^ Department of Rehabilitation Medicine Ewha Woman's University Seoul Hospital School of Medicine Ewha Woman's University Seoul Korea; ^2^ Department of Medical Statistics College of Medicine Catholic University of Daegu Daegu Korea; ^3^ Department of Physical Medicine and Rehabilitation College of Medicine Yeungnam University Taegu Korea

**Keywords:** anxiety, coronavirus, COVID‐19, depression, mental health, physical therapists

## Abstract

**Aim:**

This study aimed to explore coronavirus disease‐related psychological stress in hospital workers in nursing care hospitals during the coronavirus disease epidemic.

**Design:**

Cross‐sectional observational study.

**Methods:**

A questionnaire survey was administered to hospital workers at three nursing care hospitals.

**Results:**

Fifty‐four workers at three nursing care hospitals (9, 29 and 16 works) responded to our survey. Twenty‐four workers (50%) scored ≥5 on the Generalized Anxiety Disorder Scale, indicating the presence of anxiety. For the Patient Health Questionnaire‐9, six employees (11.1%) scored ≥10 scores, indicating the presence of depression. Workers who lived with other people with chronic underlying diseases showed significantly higher incidence of the presence of anxiety and depression. In binary logistic regression analysis, when living with persons with chronic underlying diseases, the risk of the presence of depression increased. Also, there was a higher incidence of depression in occupational therapists compared with physical therapists and nurses.

## 1 INTRODUCTION

Coronavirus disease (COVID‐19) has brought tremendous psychological stress around the world, including stress from significant morbidity and mortality (Tsou et al., 2020). The disease is caused by the severe acute respiratory syndrome coronavirus 2 (SARS‐CoV‐2) and has spread rapidly and extensively to many places outside Wuhan (Huang et al., 2020). In South Korea, the number of confirmed cases has constantly risen since the country's first confirmed case in 20 January 2020.

## 2 BACKGROUND

Hospital workers are under great physical and psychological pressure as they fight the COVID‐19 epidemic. People who work in hospitals are especially vulnerable to health care‐associated infections (Kariya, Sakon, Komano, Tomono, & Iso, 2018). During the 2003 outbreak of SARS, hospital employees reported high levels of anxiety, stress and depression (Lee et al., 2007; Wu et al., 2009) and 89% of healthcare workers reported psychological symptoms (e.g. worry over personal health and fear of social contact) (Chua et al., 2004). Currently, there are similar concerns about the psychological impact of COVID‐19 on hospital workers (Chew et al., 2020; Lai et al., 2020; Tan, Chew, et al., 2020). Hospital workers including physicians, nurses, pharmacists, technicians and clerical staff reported various psychological symptoms such as depression, anxiety, stress and psychological distress in Singapore and India (Chew et al., 2020; Tan, Chew, et al., 2020). It is important to investigate the presence of psychological problems in hospital workers during the COVID‐19 epidemic, as psychological problems may increase the risk of various disorders, such as infection, ischaemic heart disease, coronary heart disease, ventricular arrhythmia, hypertension, diabetes and even sudden cardiac death (Li, Zhang, Xiao, & Sun, 2020; Scorza et al., 2010). Psychological problems, such as major depressive disorder, were reported to increase the risk of coronary artery disease by interacting with a high triglyceride level and increasing blood pressure (Ho et al., 2018).

Coronavirus disease seems to occur more frequently in older adults and those with underlying chronic diseases. The mortality rate is especially high in older adults due to chronic illness and deterioration of the immune system (Ng et al., 2018). In particular, older adults, who live in long‐term care facilities (e.g. nursing care hospitals), are vulnerable to infection because of the characteristics of a congregate housing environment (Nikolich‐Zugich et al., 2020). Residents in long‐term care facilities are at risk of exposure to infection due to close living quarters and shared caregivers (Menec, MacWilliam, & Aoki, 2002). Thus, hospital workers working in nursing care hospitals may also be at risk for occupational exposure to COVID‐19. A recent meta‐analysis showed that healthcare workers who were COVID‐19 positive constituted a significant portion (reportedly about 10.1%) of all COVID‐19 patients (Sahu et al., 2020). A considerable risk of contracting COVID‐19 infection seems to be present among healthcare workers. Assessment of the psychological status of hospital workers in nursing care hospitals is critical to respond to this serious public health event, and this is a gap in COVID‐19 research (Tran et al., 2020). We hypothesized that the COVID‐19 epidemic may cause fear and anxiety among hospital workers, who are susceptible health care‐associated infections. Therefore, the aim of our study was to explore the psychological impact of COVID‐19 on hospital workers in nursing care hospitals.

## 3 METHODS

### 3.1 Subjects

We conducted a questionnaire survey among employees of three nursing care hospitals in South Korea, on 15 April 2020. Nursing care hospitals in Korea usually take care of patients who are admitted for convalescent rehabilitation after acute hospital care. Patients admitted to these nursing care hospitals are usually elderly, suffering from the neurological diseases such as strokes, dementias and Parkinsonism. Also, many patients have multiple chronic diseases or geriatric problems such as decreased nutritional status, depression or gait disorders. We recruited employees who had not been previously diagnosed as having a psychiatric disorder. Based on the previous study (Cuijpers, Cristea, Weitz, Gentili, & Berking, 2016), we calculated a sample size. In this study, the effect size on depression was 0.81. When we adopted type I error of 0.05, power of 80% and 2‐sided test, 25 subjects per group were found to be necessary for our study. Considering 10% as the dropout rate, we needed to recruit 56 subjects. Therefore, we recruited 56 hospital workers from three nursing care hospitals. This study was approved by the Institutional Review Board of Yeungnam university hospital (2020‐04‐089), and all participants provided written informed consent to be included in the study.

#### 3.1.1 Survey questionnaire

The questionnaire consisted of three parts: (1) epidemiology (age, sex, family living arrangement, previous medical history, level of social isolation due to contact with COVID‐19 patients and the presence of family members confirmed with COVID‐19); (2) the 7‐item Generalized Anxiety Disorder Scale (GAD‐7) (Spitzer, Kroenke, Williams, & Lowe, 2006); and (3) the Patient Health Questionnaire‐9 (PHQ‐9) (Kroenke, Spitzer, & Williams, 2001). The GAD‐7 is a tool for assessing the presence of anxiety. Total scores range from 0–21 (cut‐off value: ≥5). The PHQ‐9 is a nine‐item tool for assessing the presence of depression. Total scores range from 0–27 (cut‐off value: ≥10). Both GAD‐7 and PHQ‐9 were translated into Korean language, and the reliability and validity of GAD‐7 and PHQ‐9 have been well established (Seo & Park, 2015a, 2015b).

### 3.2 Statistical analysis

We investigated the frequency of anxiety and depression and their link to demographic variables. The chi‐square test was used to examine the association between risk of anxiety and depression and each demographic variable. If a significant association between the presence of anxiety or depression and a demographic variable was found for ≥2 categories, we conducted a binary logistic regression analysis to adjust for potentially confounding factors. Statistical significance was set at *p* < .05.

## 4 RESULTS

Two hospital workers refused to respond to our survey. Therefore, a total of 54 hospital workers from 3 nursing care hospitals (9, 29 and 16) responded to our survey. Of the 54 respondents, 11, 20, 17, 2 and 4 were occupational therapists, physical therapists, nurses, radiographers and administrative workers, respectively. Twenty‐four respondents (50%) scored ≥5 on the GAD‐7, indicating the presence of anxiety (Table 1). The mean score on the GAD‐7 was 5.1 ± 3.9. For the PHQ‐9, 6 respondents (11.1%) scored ≥10, indicating the presence of depression (Table 2). The mean PHQ‐9 score of the physical therapists was 4.7 ± 3.9. None of the 54 respondents felt that they had experienced social isolation due to contact with COVID‐19 patients or the presence of family members confirmed with COVID‐19.

**Table 1:** Associations between demographic data and the presence of anxiety in hospital workers

Variable	Total	Anxiety	No anxiety	*p*‐value
Total, *n* (%)	54	27 (50)	27 (50)	
Age range, *n*				.198
20s	20	9	11	
30s	13	4	9	
40s	11	8	3	
50s	7	5	2	
60s	3	1	2	
Sex, *n*				.000
Male	12	6	6	
Female	42	21	21	
Presence of infant or child ≤6 years of age in the home				.159
Yes	5	4	1	
No	49	23	26	
Presence of adult ≥65 years of age in the home				.150
Yes	2	2	0	
No	52	25	27	
Presence of a person with an underlying chronic disease in the home				.009*
Yes	12	10	2	
No	42	17	25	
Occupation				.053
Occupational therapist	20	5	15	
Physical therapist	11	7	4	
Nurse	19	10	7	
Radiographer	2	2	0	
Administrative workers	4	3	1	
Previous medical history				.552
Yes	3	2	1	
No	51	25	26	
Isolation experience				.587
Yes	2	1	1	
No	63	20	43	
Family members confirmed with COVID‐19				.747
Yes	4	1	3	
No	61	20	41	

Note

*p*‐values were calculated by independent *t* test or chi‐square test, as appropriate. *Significant difference noted in comparison between two groups (*p* < .05). Values are presented as mean ± standard deviation.

Previous medical history: Hypertension, 3 hospital workers.

Abbreviation: COVID‐19, coronavirus disease.

**Table 2:** Association between the presence of depression and demographic variables in hospital workers

Variable	Total	Depression	No depression	*p*‐value
Total, *n* (%)	54	6 (11.1)	48 (88.9)	
Age range, *n*				.265
20s	20	3	17	
30s	13	3	10	
40s	12	0	12	
50s	8	0	8	
60s	3	0	3	
Sex, *n*				.165
Male	12	0	12	
Female	42	6	36	
Living together with ≤6 years infant or child				.407
Yes	5	0	5	
No	49	6	43	
Living together with ≥65 years old person				.610
Yes	2	0	2	
No	52	6	46	
Living together with person with chronic underlying diseases				.005*
Yes	12	4	8	
No	42	2	40	
Occupation				.026*
Occupational therapist	11	4	7	
Physical therapist	20	1	19	
Nurse	17	0	17	
Radiographer	2	0	2	
Administrative workers	4	1	3	
Previous medical history				.529
Yes	3	0	3	
No	51	6	45	

Note

P‐values were calculated by independent *t* test or chi‐square test, as appropriate. *Significant difference noted in comparison between two groups (*p*<.05). Values are presented as mean ± standard deviation.

Previous medical history: Hypertension, 3 hospital workers.

Abbreviations: COVID‐19, coronavirus disease; ECMO, extracorporeal membrane oxygenation; FBS, fasting blood sugar; ICU, intensive care unit.

With regard to anxiety, results of the chi‐square test indicated that the hospital workers who lived with people with chronic underlying diseases, such as diabetes, hypertension, chronic kidney disease, heart disorders and stroke, showed significantly higher incidence of the presence of anxiety. Other demographic variables were not found to affect the presence of anxiety.

Regarding depression, results of the chi‐square test indicated that whether workers lived with people who had chronic underlying diseases or not and occupation were significantly related to the presence of depression. Results of the binary logistic regression analysis indicated that living with someone with a chronic underlying disease increased the likelihood of the presence of depression (Table 3). In addition, occupational therapists had higher incidence of depression than physical therapists and nurses.

**Table 3:** Binary logistic regression analysis of risk factors for depression in hospital workers

Variables	OR	95% CI for OR	*p*‐value
Presence of a person with an underlying chronic disease in the home			
Yes	7.230	1.788–29.239	.006*
No	1	–	–
Occupation			
Occupational therapist	1	–	–
Physical therapist	0.115	0.020–0.665	.016*
Nurse	0.050	0.005–0.482	.010*
Radiographer	1.317	0.104–16.709	.832
Administrative workers	0.686	0.092–5.092	.712

Abbreviations: CI, confidence interval; OR, odds ratio.

* Significant difference noted in comparison between two groups (*p* < .05).

## 5 DISCUSSION

This cross‐sectional survey investigated the psychological impact of COVID‐19 on hospital workers in nursing care hospitals. Our study revealed that 50% and 11.1% of hospital workers reported symptoms of anxiety and depression, respectively.

Transmission of COVID‐19 often occurs in the hospital setting, as the transmission of COVID‐19 frequently occurs through person‐to‐person contact and contaminated environmental surfaces (Rothan & Byrareddy, 2020). Long‐term care facilities, such as nursing care hospitals, are vulnerable to respiratory disease outbreaks, and recently, long‐term care facilities in the United States have demonstrated their vulnerability to COVID‐19 (McMichael et al., 2020). Older adults are at high risk of infection in long‐term care settings as rooms and bathrooms, which are often filled with microorganisms, are shared.

Once SARS‐CoV‐2 is introduced in a long‐term care facility, it has the tendency to spread rapidly and widely. Knowledge of this fact may cause hospital workers in nursing care hospitals to feel vulnerable to exposure to the virus. Hospital workers in nursing care hospitals provide most of daily living care for patients, which frequently involve direct contact. Many patients in nursing care hospitals are bedridden, and direct contact with these patients is almost inevitable. In addition, the closed environment of nursing care hospitals can also promote the transmission of COVID‐19 and hospital workers and patients may inadvertently carry pathogens from one person to another (Lin et al., 2011). The transmission may occur via direct contact through hands or indirect contact through air or contaminated objects (Tan, Hao, et al., 2020; Wang, Pan, Wan, Tan, Xu, McIntyre, et al., 2020).

Several previous studies have reported the psychological impact of the COVID‐19 on healthcare workers. One study (Lai et al., 2020) reported that the prevalence of depression and anxiety symptoms in healthcare workers who treated patients with COVID‐19 in China was 50.4% and 44.7%, respectively. More than 70% of healthcare workers experience psychological distress. Other recent studies (Chew et al., 2020; Tan, Chew, et al., 2020) reported the presence of depression, anxiety, stress and psychological distress in healthcare workers in Singapore and India. The most common symptom was headache, with a prevalence of 32.3% (Chew et al., 2020). The findings of our study also showed that hospital workers in nursing care hospitals had high prevalence of anxiety and depression. While caring for patients in nursing care hospitals, hospital workers may be exposed to COVID‐19, which is potentially fatal. Understanding this reality may intensify their fears and sense of danger. Among infected health workers, 14.8% have been classified as being in severe or critical condition (Wu & McGoogan, 2020), and hospital workers may be concerned about their well‐being and the health of their families (Wang, Pan, Wan, Tan, Xu, Ho, et al., 2020).

Results of the National Mental Health Survey, conducted by the South Korean government in 2016, indicate that the prevalence of anxiety and depression was 9.3% and 5.0%, respectively, among hospital workers, indicating that these workers have high rates of anxiety ("The Survey of Mental Disorders in Korea, Ministry of Health and Welfare^2016^"). Although the rate of depression was not as high as that of anxiety, it was somewhat higher than average. Our study showed that hospital workers who live with people with chronic underlying diseases are more likely to have symptoms of anxiety and depression. This finding may be due to the fact that people with underlying diseases and the elderly are at higher risk of death from COVID‐19 compared with the general population. The mortality rate was found to be highest among patients over 70 years of age with underlying diseases, such as hypertension, diabetes, asthma and renal failure (Kang, 2020). Patients with underlying diseases and older adult patients presumably have weaker immune systems that permit faster progression of viral infection; thus, these patients may be more susceptible to infection and are more at risk of critical illness (Li, Guan, et al., 2020; Wang, Tang, & Wei, 2020). This epidemiologic knowledge may affect the perspectives of hospital workers who are living with people with underlying diseases and cause them to experience more fear and anxiety about COVID‐19 than other people in other occupations.

The results of our study also showed that occupational therapists had higher incidence of depression than physical therapists and nurses. One recent study showed that the psychosocial work environment, which can include high psychological demands, low decision latitude and low social support, was more associated with depression than the physical work environment (Niedhammer, Coindre, Memmi, Bertrais, & Chastang, 2020). One possible explanation for our finding may be that occupational therapists, physical therapists and nurses have different job stressors. However, there are several limitations to this study. First, the sample size was small and there were insufficient participants representing each professional group. Caution should be exercised in generalizing the results to all hospital workers in nursing care hospitals. Further investigation with large sample size is needed. Second, the study did not include a control group nor a longitudinal follow‐up. Considering the increasing number of confirmed cases of COVID‐19, the mental health of hospital workers in nursing care hospitals could become more severe. Online cognitive behavioural therapy and mindfulness‐based therapy are helpful in treating depression and anxiety during the COVID‐19 pandemic (Ho, Chee, & Ho, 2020). It would be ideal to reinvestigate the mental health of hospital workers after a period, including long‐term courses of anxiety and depression. Third, this study was unable to distinguish pre‐existing mental health symptoms from new symptoms. Fourth, the study design did not include cause analysis for psychological strains, such as work‐related stress. Further studies considering the various factors that could affect the psychological status of hospital workers are needed in the future.

## 6 CONCLUSION

Our study has demonstrated that hospital workers in nursing care hospitals experience high levels of COVID‐19‐related psychological symptoms, including anxiety and depression. Comprehensive measures to assess and reduce the psychological stress of hospital workers are needed. Symptoms should be monitored with vigilance, and further intervention should be provided, if necessary. Additionally, hospital workers who are living with people with underlying diseases such as diabetes, hypertension, chronic kidney disease, heart disorders and stroke should be given special attention. Our study is limited in that we did not collect data on the mental health of the hospital workers who participated in our study. Similar studies that also evaluate the mental health of health workers during the COVID‐19 epidemic are warranted.

## CONFLICT OF INTEREST

The authors declared no potential conflicts of interest with respect to the research, authorship and/or publication of this article.

## Data Availability

The date sets used for the current study are available from the corresponding author on reasonable request.
